# Cardiac imaging in hypertrophic cardiomyopathy and cardiac amyloidosis: a narrative review

**DOI:** 10.3389/fcvm.2026.1752135

**Published:** 2026-03-26

**Authors:** Nicola Maggialetti, Arnaldo Scardapane, Eluisa Muscogiuri, Andrea Torrente, Giovanni Lorusso, Luca De Marco, Marco Rella, Gabriele Fanigliulo, Michele Mariano, Amato Antonio Stabile Ianora

**Affiliations:** 1 Interdisciplinary Department of Medicine, Section of Radiology and Radiation Oncology, University of Bari “Aldo Moro”, Bari, Italy; 2 Cardiac Magnetic Resonance Unit, “Perrino” Hospital, Brindisi, Italy; 3 Experimental Medicine Department, University of Salento, Lecce, Italy

**Keywords:** cardiac amyloidosis (CA), cardiovascular imaging, hypertrophic cardiomyopathy (HCM), imaging biomarkers, tissue characterization

## Abstract

Hypertrophic cardiomyopathy (HCM) and cardiac amyloidosis (CA) are major causes of myocardial thickening, yet they arise from distinct genetic, structural, and pathophysiological mechanisms. Differentiating these entities is clinically crucial, as management strategies and prognostic implications differ substantially. However, overlapping phenotypic features, particularly left ventricular hypertrophy, frequently complicate diagnosis. Recent advances in multimodality cardiac imaging have markedly improved the ability to distinguish HCM from CA through refined anatomical assessment and non-invasive tissue characterization. Echocardiography helps evaluate ventricular structure, diastolic function, and flow patterns. Asymmetric septal hypertrophy, dynamic left ventricular outflow tract (LVOT) obstruction, and systolic anterior motion (SAM) of the mitral valve are characteristic of HCM, while concentric wall thickening, biatrial enlargement, restrictive filling, and the “apical sparing” strain pattern point toward CA. Cardiac MRI adds further diagnostic value through tissue characterization. In HCM, late gadolinium enhancement (LGE) typically appears as patchy mid-wall or junctional fibrosis, whereas in CA it is diffuse, involving the subendocardium or the full wall thickness. Mapping parameters also help differentiate the two conditions: native T1 and ECV are mildly elevated in HCM but markedly increased in CA due to extensive extracellular amyloid deposition.

## 1 Introduction

Left ventricular hypertrophy represents a common final phenotypic expression of a heterogeneous group of cardiac conditions, including genetic cardiomyopathies, infiltrative diseases, pressure overload states, and metabolic or storage disorders. Within this broad spectrum of hypertrophic phenotypes, accurate etiological classification is essential, as it directly influences clinical management, prognosis, and therapeutic decision-making.

Hypertrophic cardiomyopathy (HCM) and cardiac amyloidosis (CA) are two distinct, yet clinically significant conditions, both characterized by abnormal thickening of the heart muscle. Despite substantial differences in epidemiological features, age at presentation, clinical manifestations, and disease-specific red flags, diagnostic uncertainty may arise in selected clinical scenarios, particularly in patients with unexplained left ventricular hypertrophy, atypical or early disease stages, or overlapping morphological features on initial imaging assessment.

In this context, cardiovascular imaging plays a central role in refining the diagnostic workup. Advances in echocardiography, cardiac magnetic resonance (CMR), and nuclear imaging, including single photon emission computed tomography (SPECT), have enabled a more detailed characterization of myocardial morphology, tissue composition, and functional abnormalities. These techniques provide complementary information that supports non-invasive differentiation between hypertrophic phenotypes and facilitates early diagnosis and risk stratification.

This review aims to provide a comprehensive overview of the current imaging techniques used in HCM and CA, focusing on diagnostic imaging workup and imaging biomarkers that facilitate non-invasive differentiation and prognostic risk stratification.

### 1.1 Integrated approach to cardiomyopathy diagnosis

A multiparametric and clinically oriented approach is strongly recommended for the evaluation of patients with suspected cardiomyopathy, aiming both to define the specific cardiomyopathy phenotype and to identify the underlying aetiology. This approach, often referred to as the “cardiomyopathy mindset,” emphasizes the integration of detailed clinical assessment, laboratory evaluation, and multimodality imaging in a structured, stepwise workflow. A comprehensive medical history and physical examination are essential, as age at presentation, family history, and the presence of extracardiac manifestations can provide critical diagnostic clues and suggest inherited or systemic conditions. Patients may present with a wide spectrum of symptoms, including dyspnoea, chest pain, palpitations, syncope, or presyncope, but in some cases, symptoms may be minimal or absent, highlighting the importance of careful clinical evaluation. Routine and targeted laboratory testing assists in detecting comorbidities and organ dysfunction, as well as disease-specific biomarkers such as natriuretic peptides, cardiac troponins, inflammatory markers, iron parameters, and indicators of metabolic or neuromuscular disorders. Echocardiography serves as the first-line imaging modality to evaluate ventricular morphology, wall thickness, systolic and diastolic function, and dynamic obstruction. Cardiac magnetic resonance provides comprehensive tissue characterization, including assessment of fibrosis, infiltration, or edema, while nuclear imaging techniques, such as scintigraphy and PET, are employed when specific aetiologies are suspected, enabling molecular-level assessment and quantification. The integration of clinical data, laboratory results, and imaging findings in a hierarchical, hypothesis-driven workflow facilitates accurate diagnosis, early initiation of targeted therapy, risk stratification, and longitudinal follow-up, ultimately improving patient management and outcomes (1).

## 2 HCM

HCM is clinically defined as left ventricular (LV) hypertrophy occurring in the absence of ventricular dilation and not attributable to other cardiac or systemic diseases, including abnormal loading conditions (1).

It represents one of the most prevalent genetic heart conditions, with an estimated prevalence of approximately 1 in 500 individuals. In younger populations, such as children and athletes, HCM is recognized as the leading cause of sudden cardiac death, with an annual mortality rate of roughly 1%, and nearly a quarter of patients achieve a normal life expectancy (2).

In adults, the condition typically presents with symptoms such as persistent shortness of breath, chest pain, syncope, palpitations, and heart failure (2). These manifestations are primarily related to intraventricular obstruction—most commonly at the level of the LV outflow tract (LVOT)—as well as myocardial ischemia, impaired coronary vasodilator reserve, diastolic dysfunction, and arrhythmias. However, a substantial proportion of patients may remain completely asymptomatic.

LVOT obstruction is observed in approximately 70% of HCM cases and is defined by a pressure gradient exceeding 30 mmHg, with gradients greater than 50 mmHg generally considered hemodynamically significant. When LVOT obstruction is present, the term *obstructive hypertrophic cardiomyopathy* is often used. The obstruction most commonly occurs at the basal interventricular septum (SIV) and is frequently associated with systolic anterior motion (SAM) of the chordal apparatus. Dynamic obstruction may also occur deeper within the left ventricle or at the mid-cavity level due to hypertrophic papillary muscles. Moreover, SAM of the anterior mitral leaflet can further exacerbate LVOT obstruction, thereby reducing both coronary and systemic blood flow. Secondary findings related to LVOT obstruction include mitral regurgitation, leaflet elongation or accessory tissue, left atrial dilation, and papillary muscle abnormalities such as hypertrophy, anterior displacement, or direct insertion into the anterior mitral leaflet (3, 4). Beyond the presence or absence of obstruction, HCM can present with a wide spectrum of morphological patterns, reflecting variability in the distribution and extent of hypertrophy.

### 2.1 Main phenotypes of HCM

HCM exhibits considerable morphological heterogeneity, which can be categorized into distinct phenotypic patterns based on the distribution and extent of myocardial hypertrophy. The principal morphologic variants include asymmetric, concentric (symmetrical), apical (Yamaguchi syndrome), mid-ventricular, and mass-like HCM.

**Figure 1:**
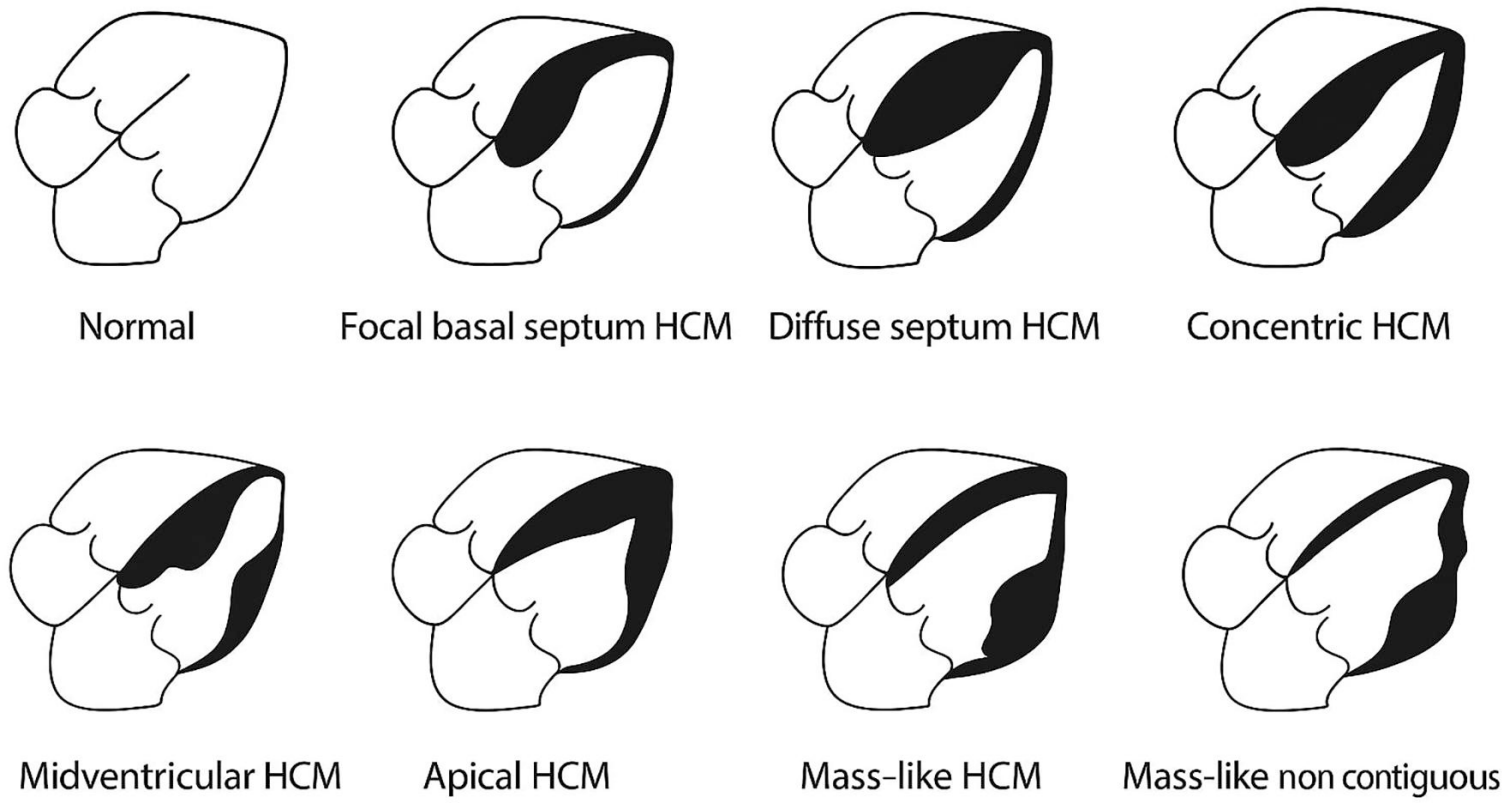
Graphical representation of HCM phenotypes.

Alternatively, a four-pattern classification model has been proposed, encompassing: hypertrophy confined to the septum (45%), hypertrophy involving the septum and additional segments with sparing of the apex (16%), hypertrophy of the apical segments in combination with other segments (27%), and isolated apical hypertrophy (13%) (5, 6) (Figure 1).

− Asymmetric HCM represents the most common phenotype, accounting for approximately 60%–70% of cases. It is characterized by disproportionate thickening of SIV, most frequently involving the basal anteroseptal and adjacent basal anterior segments. This configuration often results in narrowing of the LVOT, predisposing to dynamic obstruction. Diagnostic imaging features typically include an interventricular septal thickness ≥15 mm or values exceeding two standard deviations above the mean for age in pediatric populations.
− Symmetrical or concentric HCM is the second most frequent variant and is defined by uniform thickening of the LV wall associated with a reduction in cavity size. Secondary causes of concentric hypertrophy—such as systemic hypertension or valvular aortic stenosis—must be excluded, as well as physiological hypertrophy related to athletic conditioning (*athlete's heart*).
− Apical HCM (Yamaguchi syndrome) is characterized by predominant thickening of the apical LV segments, resulting in the pathognomonic “ace of spades” configuration of the ventricular cavity. It may present as a “mixed” form with concurrent septal hypertrophy or as a “pure” form limited to the apex. Imaging criteria suggestive of this diagnosis include an apical wall thickness ≥15 mm and an apical-to-basal wall thickness ratio >1.5.
− Mid-ventricular HCM is a less common subtype, observed in approximately 10% of patients. Hypertrophy predominantly involves the mid-cavity segments, producing an “hourglass” or “dumbbell” appearance. This pattern may lead to mid-cavity obstruction and, in some cases, to the formation of a distal apical aneurysm.
− Mass-like HCM is a rare presentation characterized by focal myocardial thickening confined to a single segment, which may mimic a neoplastic or infiltrative mass on imaging.

### 2.2 Diagnosis in HCM

#### 2.2.1 Imaging

Effective management of HCM relies on early and precise diagnosis. The absence of a definitive diagnostic test, the broad spectrum of phenotypic variations, the frequent occurrence of phenocopy conditions, and the presence of normal or hyperdynamic LV function in most patients make HCM a condition that heavily relies on imaging. Imaging plays a crucial role in all aspects of management, including diagnosis, classification, risk assessment for complications, complication detection, evaluation of risk for ventricular arrhythmias, therapy selection and monitoring, intraprocedural guidance, and family screening. While echocardiography remains the cornerstone for diagnosing and managing HCM, this condition clearly necessitates the use of multiple imaging techniques to ensure comprehensive and optimal patient care (7) (Table 1).

**Table 1:** Imaging features for differential diagnosis between HCM and CA.

Feature	HCM	CA
Pattern of hypertrophy	Asymmetric (often basal septal); may be apical, mid-ventricular, or concentric	Typically concentric and symmetric; occasionally asymmetric (especially ATTR)
LV wall thickness	≥15 mm; localized hypertrophy	>12 mm; diffuse and homogeneous thickening
LV cavity	Small or normal	Small, with restrictive physiology
LVOT obstruction/SAM	Common (∼70%), dynamic obstruction with SAM of mitral valve	Absent
Diastolic function	Impaired relaxation, pseudonormal or restrictive in advanced stages	Restrictive filling
		Echocardiography: E/A > 2, short deceleration time
Atrial findings	Left atrial enlargement common	Biatrial enlargement typical
Echocardiography Myocardial texture	Echocardiography: Homogeneous echogenicity; CMR: normal or mildly increased T1/ECV	Echocardiography: “Granular sparkling” appearance;
		CMR: markedly elevated T1/ECV due to amyloid infiltration
Strain pattern	Reduced longitudinal strain in hypertrophied segments; heterogeneous pattern	Characteristic “apical sparing” (basal-to-apical strain gradient)
CMR Myocardial features	− Patchy mid-wall LGE or junctional fibrosis, often at RV insertion points − Mildly elevated T1; normal or mildly increased T2	− Diffuse subendocardial or transmural LGE; difficulty in myocardial nulling (“dark blood pool” sign) − Markedly increased native T1 and ECV (>40%); elevated T2 (expecially in AL subtype)
SPECT	Perfusion imaging to detect microvascular ischemia and prognostic stratification using ⁹⁹mTc-sestamibi or tetrofosmin	Bone-avid tracer scintigraphy for non-invasive ATTR diagnosis using ⁹⁹mTc-PYP, ⁹⁹mTc-DPD, or ⁹⁹mTc-HMDP (Perugini score ≥2)
PET	Quantitative assessment of myocardial blood flow and coronary flow reserve with ¹³N-ammonia or ⁸²Rb, providing prognostic information	Amyloid-targeted PET for direct visualization and quantification of myocardial amyloid using ¹¹C-PiB or ¹⁸F-labeled tracers
		Primarily used to differentiate AL from ATTR cardiac amyloidosis
Pericardial effusion	Rare	Common
Right ventricular involvement	Uncommon	Frequent; RV wall thickening
		Echocardiography: reduced TAPSE/S’

#### 2.2.2 Diagnostic criteria

According to the ESC Scientific Document Group's 2023 ESC Guidelines for the Management of Cardiomyopathies (8), the diagnosis of HCM is based on specific criteria depending on the age group and familial context (Table 2). In adults, HCM is defined by a left ventricular (LV) wall thickness of 15 mm or more in any myocardial segment, provided that the hypertrophy cannot be fully explained by abnormal loading conditions. When wall thickness is between 13 and 14 mm, the diagnosis requires additional supporting elements, such as a positive family history, the presence of disease-causing genetic variants, or electrocardiographic abnormalities.

**Table 2:** HCM and CA diagnostic criteria according to ESC (8).

HCM	CA
LV wall thickness ≥15 mm, not explained by abnormal loading	Positive cardiac biopsy
or	Or
LV wall thickness 13–14 mm **+** family history, pathogenic genetic variant, or ECG abnormalities	Positive extracardiac biopsy + Echocardiographic/CMR features (Supplementary Table S1)
Or	Only ATTR:
	Absent monoclonal protein + Grade 2 or 3 cardiac uptake at scintigraphy + Echocardiographic/CMR features
LV wall thickness ≥ 13 mm + first-degree relatives	
Children:	
LV wall thickness >2 SD above predicted mean (z-score > 2)	

In children, HCM is diagnosed when the LV wall thickness exceeds two standard deviations above the predicted mean for age and body size (z-score >2). For first-degree relatives of patients with confirmed HCM, the diagnostic threshold in adults is an LV wall thickness of 13 mm or greater. In pediatric first-degree relatives, a z-score below 2 does not confirm the diagnosis, but the presence of associated morphological features or ECG abnormalities should raise clinical suspicion, even if these findings alone are not sufficient for a definitive diagnosis.

#### 2.2.3 Echocardiography

As increased ventricular wall thickness can be found at any location (including the right ventricle), the presence, distribution, and severity of hypertrophy should be documented*.* Two-dimensional echocardiography provides a comprehensive view of the morphological and functional characteristics of HCM, including ventricular wall hypertrophy, SAM, ventricular systolic function, and valve assessment in the right ventricle. The addition of color Doppler and spectral Doppler further enhances the ability to noninvasively measure LVOT gradients and assess mitral regurgitation both qualitatively and quantitatively, allowing for more precise differentiation of HCM (9).

Pulsed wave Doppler (PWD) may be used to differentiate obstruction occurring at the mid-cavitary level, in the outflow tract, or at the level of the aortic valve by placing the “gate” and obtaining spectral envelopes from the corresponding locations. Aliasing from high velocity flow is typical of obstruction.

Continuous wave Doppler (CWD) envelope morphology can suggest the nature (i.e., fixed vs. dynamic) of the obstruction and may be used to quantify the peak gradient as a surrogate of hemodynamic significance.

Dynamic obstruction demonstrates an envelope with a gradual velocity increase in early systole followed by a sharp increase heralding the onset of obstruction with a late peak. A peak gradient (4 × peak velocity 2) of <30 mmHg is unlikely to be hemodynamically significant (10).

In addition, Transesophageal echocardiography (TEE) provides the benefit of being closer to the heart, without intervening structures, allowing for high-quality imaging. TEE can be utilized in cases of HCM when transthoracic images are of insufficient quality.

Stress echocardiography provides several advantages in evaluating patients with HCM. It allows clinicians to assess exercise tolerance, measure LVOT gradients under stress, and identify inducible ischemia. As patients often experience symptoms during physical activity, imaging during exercise offers valuable insights. While treadmill testing is the most commonly used approach, the supine bicycle is also utilized. One benefit of the supine bicycle is that it enables imaging throughout the different stages of exercise, in contrast to treadmill testing, where imaging is usually done only after exercise has finished (11).

Strain echocardiography is a sensitive, quantitative, and well-validated method for assessing both segmental/regional and global cardiac mechanics. It works by evaluating the rates and extent of segmental and global shortening and lengthening. While there are specific regional patterns of strain abnormalities, the majority of research has concentrated on measuring global longitudinal strain (GLS) (12).

Although single photon emission computed tomography (SPECT) is almost always the initial technique for a nuclear imaging assessment of myocardial perfusion, cardiac positron emission tomography (PET) offers advantages over SPECT, such as higher spatial resolution, superior detection sensitivity, better temporal resolution, and improved correction methods for photon scatter and photon attenuation. 13N-ammonia (13NH3) and 15Owater have superior pharmacokinetic properties because of a greater myocardial net uptake rate at higher coronary flows compared with their SPECT counterpart. PET allows data acquisition in dynamic sequences to delineate tracer kinetics and enables quantification of myocardial blood flow (11).

#### 2.2.4 Cardiac magnetic resonance

Although TTE is an essential tool for the initial evaluation of patients with left ventricular hypertrophy, it has limitations, particularly in cases of suboptimal echocardiographic windows. CMR plays a critical role in providing anatomical details and myocardial tissue characterization, enabling the accurate classification of these patients based on their phenotype. CMR is recommended in patients with HCM at their baseline assessment (8).

The recommended CMR protocol includes: breath-hold cine balanced steady-state free procession images (b-SSFP) for morphological assessment and for quantifying ventricular volumes, ejection fraction, and mass; native and post-contrast T1 mapping with extracellular volume (ECV) measurement (T1 map acquisition is recommended in two short-axis slices and a 4-chamber view before and after contrast); early and Late Gadolinium Enhancement (EGE and LGE) (13).

Standardized cine two-, three-, and four-chamber SSFP planes offer basical morphological insights; LVOT plane can also reveal turbulent jets across the LVOT in patients with HCM, aiding in the precise localization of the flow obstruction site. Furthermore, SSFP CMR can identify other abnormalities associated with HCM, such as congenital ventricular outpouchings (e.g., recesses, diverticula, aneurysms, clefts, and crypts), anomalies of the mitral valve apparatus, and abnormalities in the papillary muscles. LGE imaging provides non-invasive tissue characterization, identifying interstitial and replacement fibrosis associated with HCM. LGE is present in 65% of patients (range 33%–84%), typically in a patchy mid-wall pattern in areas of hypertrophy and at the anterior and posterior RV insertion points (14). LGE is unusual in non-hypertrophied segments except in advanced stages of disease, when full-thickness LGE in association with wall thinning is common. The absence of fibrosis may be helpful in differentiating HCM from physiological adaptation in athletes, but LGE may be absent in people with HCM, particularly young people and those with mild disease (15).

As noted above, certain features of LGE may have prognostic value. Studies have shown that the “grey zone” of fibrosis—also called “intermediate-signal LGE” or “mild-enhancement”—better predicts ventricular tachycardia risk than gross hyper-enhanced scar, likely due to viable, stressed myocytes interspersed within small scar or plexiform fibrosis. Assessing mild-enhancement LGE is challenging, as the signal intensity threshold separating mild from hyper-enhancement is arbitrary. Recently, LGE dispersion mapping was introduced: for each LGE voxel, the signal of surrounding voxels is evaluated and scored from 0 (all similar) to 8 (all different). This yields a global dispersion score (GDS), reflecting myocardial LGE heterogeneity. High GDS is associated with worse prognosis and adds predictive value beyond LGE extent for major arrhythmic events (16).

T1-weighted multi-slice gradient-echo first-pass gadolinium perfusion imaging, either at rest or under pharmacological stress, can detect ischemic segments caused by blood supply imbalance in hypertrophied areas. Native T1 values are elevated in HCM and show a correlation with wall thickness. This relationship suggests that native T1 can serve as a marker of disease severity (17, 18).

Patients with HCM exhibit reduced post-contrast myocardial T1, consistent with the presence of diffuse interstitial fibrosis in regions outside areas of LGE. ECV in HCM [29.1 ± 0.5% (1.5 T)] measured in segments without LGE, has been reported to fall at the upper limit of the normal range for healthy individuals (19).

ECV can assist in distinguishing HCM from athletic remodeling in athlete's heart, particularly in individuals within the grey zone of LV wall thickness (12–15 mm). While ECV increases with greater LV hypertrophy in HCM (due to extracellular matrix expansion and myocardial disarray), it decreases in athletes as wall thickness increases (due to cellular hypertrophy and the accumulation of healthy myocardium) (20).

However, the influence of myocardial disarray on T1 mapping in HCM remains debated, potentially leading to an overestimation of ECV (21).

Strain CMR provides a highly reproducible and operator-independent assessment of myocardial deformation, complementing conventional functional parameters such as ejection fraction. Using techniques such as feature tracking (FT-CMR) or tagging, CMR strain analysis enables quantitative evaluation of global and regional myocardial mechanics, including longitudinal, circumferential, and radial strain. It allows early detection of subtle myocardial dysfunction, even in segments with preserved wall motion or normal wall thickness, and offers valuable insights into disease progression and response to therapy.

**Figure 2:**
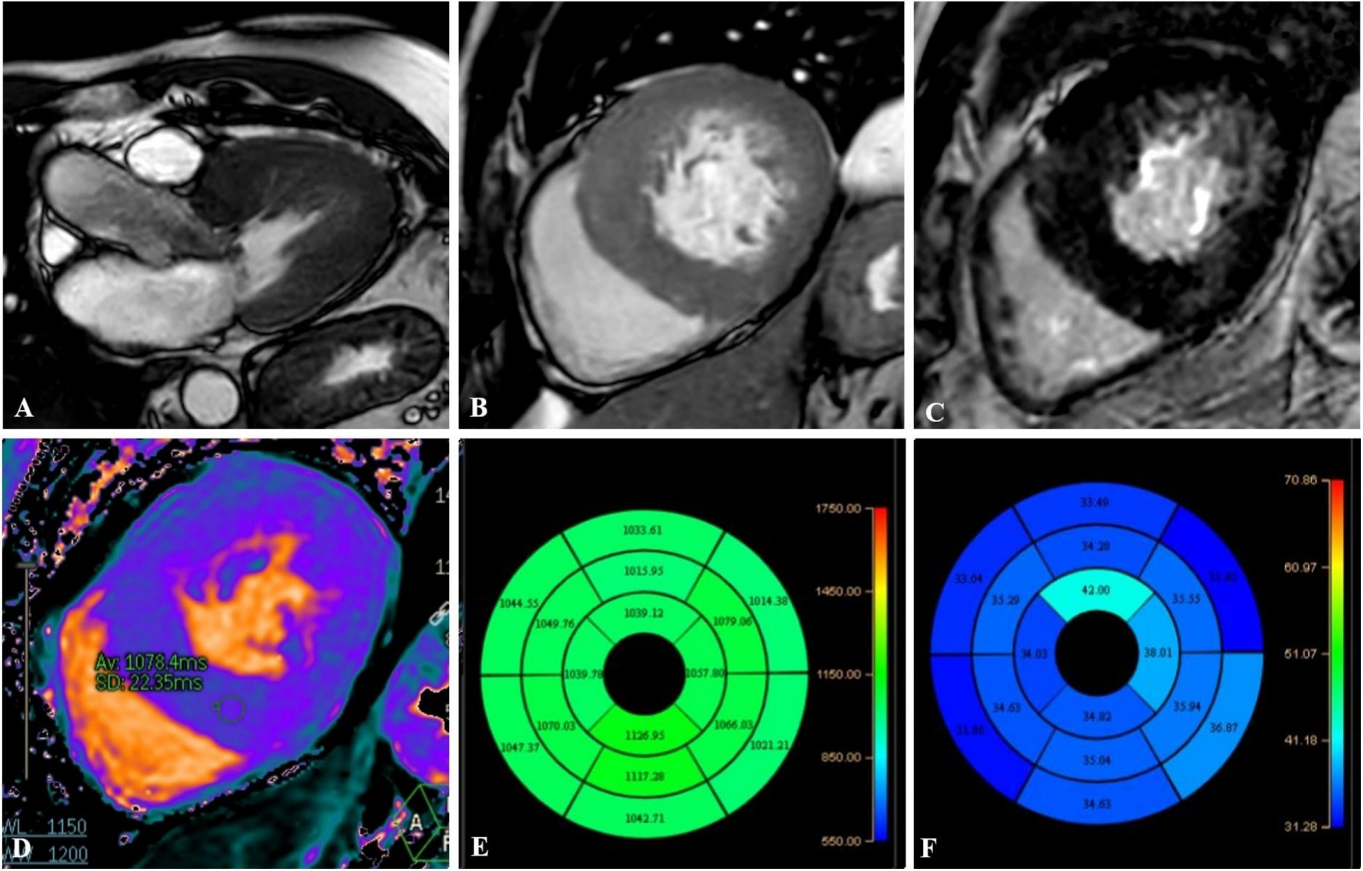
A 45-year-old patient with asymmetric septal hypertrophy. balanced-steady state free precession (b-SSFP) three-chamber **(A)** and short-axis **(B)** shows asymmetric septal thickening of the left ventricle with a maximum myocardial thickness of 25 mm. PSIR short-axis sequence **(C)** shows patchy intramyocardial LGE. T1 native Mapping **(D)**, bullseye map of T1 Native **(E)** and ECV **(F)** demonstrates increased intramyocardial value in the left ventricular walls.

**Figure 3:**
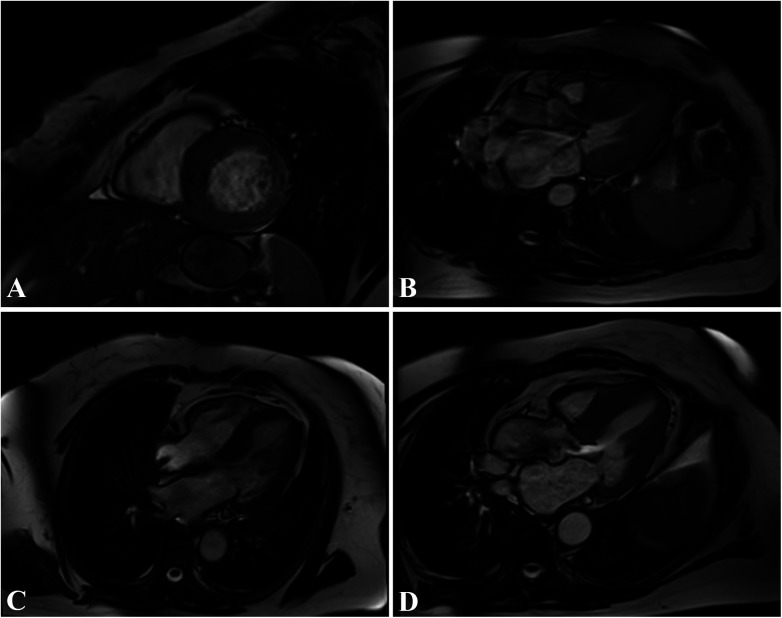
58-year-old patient with asymmetric apical hypertrophy. Four-chamber balanced-steady state free precession (b-SSFP) MRI **(A)** shows asymmetric apical thickening of the left ventricle with maximum myocardial thickness of 22 mm. PSIR sequence highlights patchy intramyocardial LGE in four-chamber **(B)** and two-chamber **(C)** T1 native Mapping **(D)** demonstrates increased intramyocardial value in the left ventricular walls.

**Figure 4:**
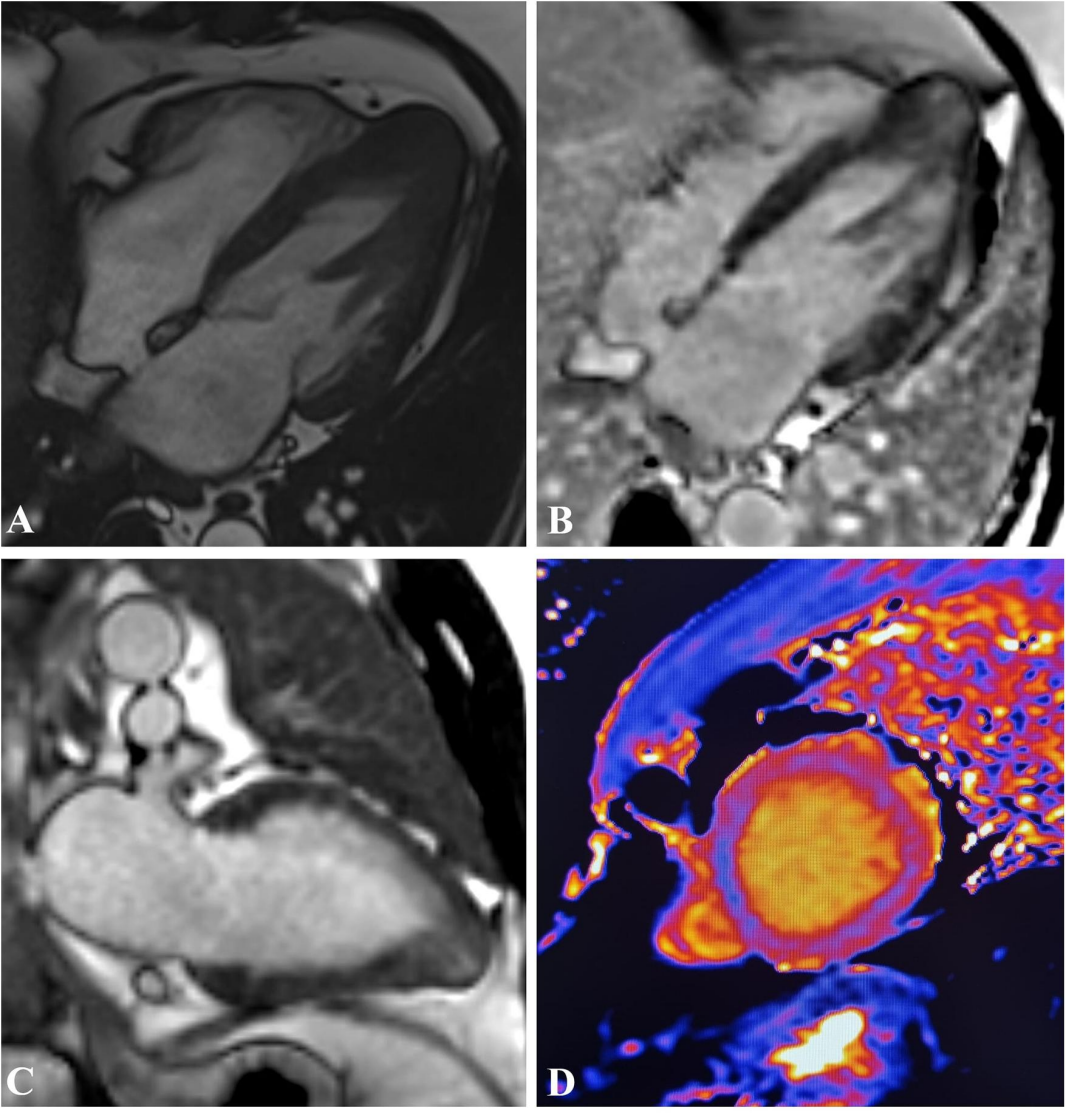
A 47-year-old patient with obstructive septal hypertrophy. The short-axis **(A)**, three-chamber **(B,D)**, and four-chamber **(C)** balanced free precession (b-SSFP) MRI sequence shows asymmetric septal thickening of the left ventricle with a maximum myocardial thickness of 24 mm and left ventricular outflow tract obstruction.

As in echocardiography, GLS has emerged as the most widely studied and clinically relevant parameter, demonstrating strong correlations with myocardial fibrosis, adverse remodeling, and major cardiac events in patients with HCM (21) (Figures 2–4).

### 2.3 Clinical outcomes and prognostic indicators

Although most individuals with HCM experience a normal lifespan, they remain at risk for serious complications, including heart failure and sudden cardiac death. Fatal outcomes are most often linked to ventricular arrhythmias, such as ventricular fibrillation. Multimodality imaging features associated with poor prognosis in HCM encompass a range of findings across echocardiography and CMR. On echocardiography, markers of adverse outcome include severe left ventricular diastolic dysfunction, a markedly elevated LVOT peak gradient exceeding 80 mmHg, and pronounced left ventricular hypertrophy. CMR provides complementary prognostic information, with features such as LGE exceeding 15%, pronounced heterogeneity of LGE—often semi-quantified through dispersion mapping—left ventricular wall thickness of 30 mm or greater, the presence of left ventricular apical aneurysms, and reduced left ventricular ejection fraction below 50% all being associated with a higher risk of adverse events (10, 22).

In adult patients with HCM, studies report an annual cardiovascular death rate of 1%–2%, with sudden cardiac death (SCD), heart failure, and thromboembolism being the primary causes. The most common fatal arrhythmia is spontaneous ventricular fibrillation (VF), though asystole, AV block, and pulseless electrical activity are also observed. Left ventricular apical aneurysms, a distinct thin-walled segment at the apex of the left ventricle, are found in about 3% of HCM patients and may be linked to an increased risk of SCD. The Task Force recommends individualized decisions for ICD implantation based on established risk factors, not just the presence of an apical aneurysm. Patients with left ventricular systolic dysfunction (LVSD) have a significantly higher incidence of SCD (7%–20%) compared to those with normal LV function and the presence of an LV ejection fraction (LVEF) <50% should then be considered in shared decision-making regarding prophylactic ICD implantation. LGE, when extensive, is associated with higher SCD risk. For patients at low to intermediate risk, the presence of extensive LGE (≥15%) can be factored into decisions about ICD implantation (8).

## 3 Cardiac amyloidosis

Cardiac amyloidosis is a pathological condition characterized by the extracellular deposition of amyloid fibrils within the myocardial tissue. These fibrils, formed from precursor proteins that undergo misfolding and aggregate into insoluble beta-sheet structures, disrupt the architecture and function of the heart. The condition often presents with myocardial thickening, leading to impaired compliance, diastolic dysfunction, and progressive heart failure (23, 24).

### 3.1 Pathophysiology in CA

Over 30 proteins are known to form amyloid aggregates *in vivo*, but nine amyloidogenic proteins mainly accumulate in the heart, causing significant cardiac disease. Currently, more than 98% of diagnosed cardiac amyloidosis (CA) is due to fibrils composed of monoclonal immunoglobulin light chains (AL) or transthyretin (ATTR), a serum transport protein for thyroid hormone and retinol, primarily produced by the liver. ATTR amyloidosis is further divided into hereditary (ATTRv) and acquired (ATTRwt or wild-type) forms. However, other types, such as AApoAI, AApoAII, AApoAIV, Ab2M, AFib, and AGel, are extremely rare. CA secondary to chronic inflammatory and infectious diseases (AA) is now less common, although still observed (24). Cardiac involvement is frequent in systemic AL amyloidosis, affecting up to 75% of patients, whereas in ATTR amyloidosis, it is the dominant clinical manifestation (25).

The deposition of amyloid within the myocardial extracellular matrix (ECM) exerts multiple deleterious effects. Accumulation of amyloid fibrils increases myocardial thickness and stiffness, leading initially to diastolic dysfunction. In advanced stages, amyloid deposits can impair electromechanical coupling and myocardial perfusion, ultimately resulting in systolic dysfunction. Both ECM remodeling and amyloid accumulation may also provoke an inflammatory response (26).

### 3.2 Diagnosis in CA

#### 3.2.1 Imaging

Cardiac amyloidosis imaging refers to the various diagnostic imaging techniques used to assess and diagnose amyloid deposition in the heart including echocardiography (often the first imaging test used to assess heart structure and function), CMR (highly sensitive for detecting amyloid deposits), myocardial scintigraphy like 99mTc-pyrophosphate (can detect amyloid deposits in the heart), positron Emission Tomography (PET scans with amyloid-specific tracers, such as 18F-florbetapir or 18F-florbetaben, are used to detect and localize amyloid deposits) and endomyocardial biopsy (gold standard for confirming the presence of amyloid deposits). These imaging techniques, combined with clinical and laboratory tests, help in diagnosing and staging cardiac amyloidosis, guiding treatment decisions, and monitoring disease progression (24) (Table 1).

#### 3.2.2 Diagnostic criteria in CA

CA is diagnosed with certainty when amyloid fibrils are found within cardiac tissue; endomyocardial biopsy and immunohistochemistry tests are still considered the diagnostic gold standard. Invasive diagnostic criteria apply to all forms of CA, whereas non-invasive criteria are accepted specifically for ATTR (25).

CA is confirmed through endomyocardial biopsy, which demonstrates amyloid deposits upon Congo red staining. Following the identification of amyloid, classification of the amyloid fibril protein is performed. Although mass spectrometry is considered the gold standard for defining the type of amyloid, immunohistochemistry or immunoelectron microscopy is routinely used for amyloid typing in specialized centers. According to ESC (27), DGK (28), and CCS/CHFS (29), diagnosis can also be confirmed if amyloid deposits within an extracardiac biopsy are accompanied either by characteristic features of CA by echocardiography (in the absence of an alternative cause for increased LV wall thickness) or by characteristic features on CMR.

Cardiac ATTR amyloidosis can be diagnosed even without histological confirmation when typical echocardiographic/CMR findings are present along with scintigraphy using bone radiotracers such as 99mTc-pyrophosphate (PYP), 99mTc-3,3-diphosphono-1,2-propanodicarboxylic acid (DPD) or 99mTc-hydroxymethylene diphosphonate (HMDP) demonstrating grade 2 or 3 myocardial uptake. Additionally, exclusion of clonal dyscrasia is required through serum free light chain (FLC) assay, serum (SPIE), and urine (UPIE) protein electrophoresis with immunofixation (27).

Therefore, in the presence of echocardiographic/CMR findings with grade 2/3 of myocardial scintigraphy uptake in the absence of a clonal abnormality is highly specific to diagnose ATTR cardiac amyloidosis avoiding the need for endomyocardial biopsy. In cases of confirmed ATTR CA, genetic counselling with TTR gene sequencing is recommended to differentiate between ATTRwt and ATTRv forms (Table 2).

#### 3.2.3 Ecocardiography

Echocardiography is a fundamental tool in the study of cardiac amyloidosis, allowing the identification of structural and functional features characteristic of this infiltrative disease. Transthoracic echocardiography findings in cardiac amyloidosis include several characteristic features. Atrial septal thickening is commonly observed and is considered a hallmark of the condition. The left ventricular (LV) myocardium often displays a granular or sparkling appearance, though this is nonspecific and requires differentiation from other infiltrative diseases. Increased LV wall thickness results from amyloid infiltration of the interstitial space and may correlate with amyloid burden. Despite near-normal LVEF, decreased LV end-diastolic volume leads to reduced stroke volume, while systolic dysfunction may occur in advanced stages of the disease.

Transmitral flow patterns typically demonstrate a restrictive filling profile, with an exaggerated E/A ratio (E ≫ A) and a truncated E wave deceleration time. A reduced A wave amplitude suggests impaired atrial function, increasing the risk of thrombus formation. An elevated E/e′ ratio indicates raised left atrial pressure. Pulmonary venous Doppler findings often show diastolic filling predominance, with an S/D ratio <1, an atrial reversal (AR) amplitude >35 cm/s, and an AR duration exceeding the mitral A wave by more than 20 ms (30).

Biatrial enlargement, including right and left atrial enlargement, is common, often accompanied by significantly reduced atrial strain. Impaired longitudinal strain (LS) in the LV is typically more pronounced at the base and mid-ventricular regions compared to the apex. This specific strain pattern may help differentiate cardiac amyloidosis from aortic stenosis and HCM. Additionally, reduced right ventricular (RV) systolic excursion velocity (S') measured via tissue Doppler imaging and decreased tricuspid annular plane systolic excursion (TAPSE) are early indicators of cardiac involvement in systemic AL amyloidosis, despite a normal RV end-diastolic dimension (8).

Several validated echocardiographic scoring systems have been developed to improve the differential diagnosis of cardiac amyloidosis and to distinguish it from other causes of left ventricular hypertrophy. The Increased Wall Thickness (IWT) score is a multiparametric tool derived and validated in multicenter cohorts, integrating relative wall thickness, E/e′ ratio, longitudinal strain, and selected clinical features; it has demonstrated high diagnostic accuracy for cardiac amyloidosis in patients with increased LV wall thickness and preserved or mildly reduced LVEF (31). Similarly, the AMYLI score combines interventricular septal thickness, diastolic dysfunction parameters, and relative apical sparing on longitudinal strain analysis, allowing effective differentiation between cardiac amyloidosis and hypertrophic cardiomyopathy in patients with unexplained LV hypertrophy (32). More recently, the Mayo-ATTR score was specifically developed to identify transthyretin cardiac amyloidosis, incorporating age, LV wall thickness, diastolic indices, and strain-derived parameters. This score has shown robust performance in distinguishing ATTR amyloidosis from both AL amyloidosis and non-amyloid hypertrophic phenotypes, supporting its use as a screening tool in clinical practice (33).

#### 3.2.4 Nuclear imaging

Nuclear medicine plays a central and expanding role in the diagnostic workup of cardiac amyloidosis, offering highly specific molecular imaging techniques that enable non-invasive tissue characterization. Bone-avid technetium-99m–labeled radiotracers, including ^99mTc-PYP, ^99mTc-DPD, and ^99mTc-HMDP, are the mainstay of scintigraphic evaluation and have demonstrated excellent diagnostic performance for ATTR-CA. Visual assessment is commonly performed using the Perugini score, which grades myocardial uptake relative to bone activity on planar images from grade 0 (no cardiac uptake) to grade 3 (cardiac uptake greater than bone). A Perugini score ≥2, in the absence of a monoclonal protein, is considered diagnostic for ATTR-CA, with reported sensitivity exceeding 99% and specificity approaching 100%. SPECT imaging further improves anatomical localization and helps distinguish true myocardial uptake from blood pool activity. Semi-quantitative indices, such as the heart-to-contralateral lung ratio, enhance reproducibility and allow assessment of amyloid burden. Beyond scintigraphy, several PET radiotracers have been investigated for the diagnosis, risk stratification, and monitoring of CA. [11C]PiB, a thioflavin T–derived tracer with a short half-life of 20.4 min requiring an on-site cyclotron, is not FDA-approved but has demonstrated high specificity for detecting myocardial amyloid in both AL- and ATTR-CA, with higher uptake in AL-CA. Combined use with [99mTc]PYP scintigraphy can differentiate AL- from ATTR-CA, and [11C]PiB uptake correlates with histological amyloid burden and native T1 mapping on PET/MRI. Higher myocardial uptake independently predicts worse outcomes in AL-CA, and case reports suggest potential utility for tracking therapy response, although systematic studies are lacking.

Among ^18F-labeled tracers, [18F]Flutemetamol shares structural similarities with [11C]PiB, has a 110-minute half-life, and is FDA-approved. It shows uptake in CA patients with higher retention in AL-CA, and sensitivity and specificity for ATTRv-CA have been reported at 88% and 100%, respectively. [18F]Florbetapir, also FDA-approved, exhibits higher myocardial uptake in CA patients, with a trend toward greater retention in AL-CA. It may assist in risk prediction, including early detection of right ventricular involvement, and small studies suggest potential use in monitoring therapy, though larger studies are needed. [18F]Florbetaben, an ^18F-labeled stilbene, similarly shows higher uptake in AL-CA vs. ATTR-CA, and dynamic or delayed acquisitions improve discrimination between subtypes. Semi-quantitative metrics such as myocardial tracer retention correlate with echocardiographic parameters and may predict all-cause mortality in AL-CA.

[18F]-NaF, originally developed for bone imaging, has been repurposed for CA to differentiate ATTR- from AL-CA based on myocardial calcium handling. ATTR-CA shows higher myocardial uptake with faster kinetics and patchy distribution, and quantitative metrics like target-to-background ratio improve discrimination, especially when combined with CMR.

[124I]Evuzamitide is a pan-amyloid PET tracer with a 4.2-day half-life and FDA breakthrough therapy designation, binding all major amyloid types, including AL, ATTRv, and rare subtypes. Early human studies demonstrate high sensitivity for detecting cardiac and systemic amyloid, with predominant uptake in the left ventricle and correlations with echocardiographic and MRI parameters. Compared with [18F]Florbetapir, it shows similar performance in AL-CA and potentially superior application in ATTR-CA. [124I]Evuzamitide PET holds promise for diagnosis, quantification of amyloid burden, and therapy monitoring, though its prognostic value requires further investigation (34).

#### 3.2.5 Cardiac magnetic resonance

CMR provides highly accurate and detailed characterization of cardiac tissue and morphology, playing a crucial role in functional assessment, providing a fundamental tool to distinguish CA from other hypertrophic phenocopies (35).

A comprehensive CMR evaluation for CA includes morphologic and functional assessment of the left and right ventricles and atria.

The Society of Cardiovascular Magnetic Resonance (SCMR) and the European Association of Cardiovascular Imaging (EACVI) strongly recommend a standardized protocol including breath-hold cine balanced steady-state free procession images (b-SSFP), native and post-contrast T1 mapping with ECV measurement (T1 map acquisition is recommended in two short-axis slices and a 4-chamber view before and after contrast), and EGE and LGE (13).

CA morphologic and functional assessment shows typical features of restrictive cardiomyopathy:

LV global wall thickening (>12 mm), predominantly at the basal segments, typically, concentric in AL but can be asymmetric in ATTR CA; preserved or reduced LV systolic function (LV ejection fraction < 60%) with possible apical functional sparing in advanced case; reduced end-diastolic volume (<90 mL) with a reduced LV stroke volume index (<35 mL/m^2^); biatrial enlargement (left atrium > 41 mm, right atrium >44 mm); atrial septum thickening (≥6 mm); and pericardial and pleural effusion (36, 37).

In case of CA we observe the typical different contractility of apical and basal portions of left ventricle, typical of ATTR CA, with very early alterations such as reduction of longitudinal strain at the level of the basal segments with typical apical sparing (38).

Native T1 values (pre-gadolinium contrast) that provide a combined signal from myocyte and extracellular space are increased in areas of amyloid deposition in both ATTR and AL patients compared with normal and HCM tissues; increased native T1 values could allow characterization and detection of the degree of infiltration (25). In AL CA, T1 values are higher than ATTR CA (37). A positive correlation between T1 values and CMR indexes of systolic and diastolic dysfunction was observed (39).

According to EACVI, patients with CA could have T1 values >1,050–1,150 ms. Native T1 may find utility in cases when the administration of contrast is contraindicated. ECV measurement enables to isolate and quantify the signal from the extracellular space. The reference value for ECV is 23%–28%. Native T1 and ECV demonstrate correlations with CA severity and outcomes, creating anticipation for their application in the assessment of risk and therapeutic effects and progress monitoring and other aspects of clinical management (40). ECV values are higher in ATTR than in AL CA, in which histology shows myocyte hypertrophy (41, 42). Unfortunately, ECV values in CA overlap with other cardiomyopathic pathologies such as acute myocardial infarction (AMI), HCM, dilated cardiomyopathy (DCM), etc (43). Although the values of ECV are not specific, EACVI and AHA indicate that values of ECV > 40% are highly suggestive of CA. Recent studies have demonstrated that ECV results elevated in the earliest phases of the disease highlight the potential role of ECV as an early disease marker (25).

Ridouani et al. reported that myocardial native T2 values were significantly higher in AL (63.2 ± 4.7 ms) than in ATTR (56.2 ± 3.1 ms) patients and both higher than in healthy subjects (51.1 ± 3.1 ms). Nevertheless, a substantial overlap in mean T2 values between CA and control groups has been observed (44).

Although quantitative imaging parameters offer important diagnostic support, their absolute cut-off values should be applied with caution, given their dependence on technical settings, acquisition protocols, and post-processing methodologies.

Traditional LGE imaging techniques require an operator-determined null point, which is the inversion recovery time at which the normal myocardium appears black or “nulled”. This can be challenging in CA: myocardium null together or subsequently to blood pool due to expansion of the extracellular myocardial volume (from amyloid infiltration); this phenomenon, known as dark blood pool, is highly sensible and moderately specific for CA (25).

LGE reflects both amyloid deposition in the myocardial interstitium and subendocardial ischemic changes (fibrosis) associated with microangiopathy. Although multiple LGE distributions have been described in CA, LGE has almost a pathognomonic distribution:diffuse, subendocardial, and/or transmural (45). LGE may be also present in the RV wall, LA wall, and atrial septum. Subendocardial LGE is more prevalent in AL CA while transmural LGE is more prevalent in ATTR CA (46). LGE is highly prevalent (96%–100%) and more common in ATTR than AL CA but cannot distinguish between the subtypes (47). It has been noticed that LGE is a significant predictor of mortality both in AL and ATTR (46).

Zhao et al. meta-analysis based on seven published studies, estimated a sensitivity of 85% and a specificity of 92%, for CMR-based LGE in diagnosing CA (48). Vermes E et al. have highlighted a base-apex LGE gradient (49), whereby LGE mostly occurs in the basal segments.

**Figure 5:**
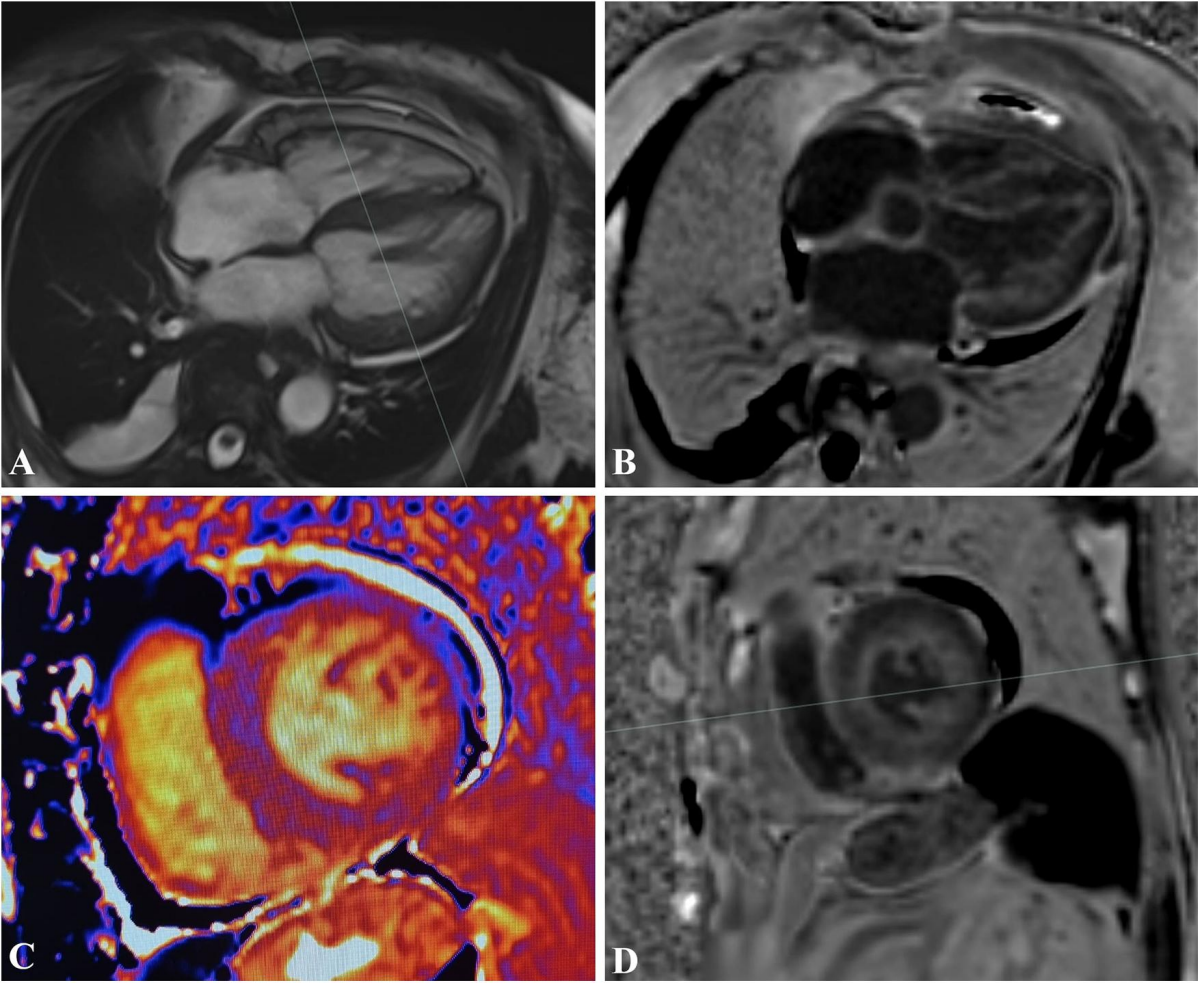
61-year-old patient with cardiac amyloidosis. Four-chamber balanced-steady state free precession (b-SSFP) MRI **(A)** shows thickening of the left ventricle. PSIR sequence highlights circumferential subendocardial LGE of left and right ventricular walls in four-chamber **(B)** and short-axis **(D)** T1 native Mapping **(D)** demonstrates increased intramyocardial value in ventricular walls.

Dungu et al. (50) proposed the Query Amyloid Late Enhancement (QALE) score to identify and study the different LGE patterns in CA. The QALE score appears to have a discriminating ability in differentiating types of amyloidosis, particularly regarding the AL form vs. other forms, such as ATTR, and provides a powerful independent prognostic value in patients with AL amyloidosis. However, QALE score requires further validation in larger prospective studies (24) (Figure 5).

### 3.3 Prognostic indicators

Both AL and ATTR cardiac amyloidosis (CA) significantly affect patients' quality of life and outcomes, making cardiac assessment essential for risk stratification and treatment decisions. Imaging plays a critical role in determining risk levels in patients with CA. Several CMR measures are prognostically significant in CA, including the presence and pattern of LGE, native T1, post-contrast T1, native T2 and various morphologic parameters (51).

A reduced LV ejection fraction, the presence of right ventricular LGE, right ventricular ejection fraction (RVEF), and TAPSE are all linked to higher mortality.

While early reports on the prognostic value of LGE in cardiac amyloidosis were conflicting, recent studies have shown that the LGE pattern can indeed serve as an independent prognostic factor when adjusted for echocardiographic findings and blood biomarkers (NT-proBNP and troponin) (52).

The LGE pattern holds prognostic value in both AL and ATTR CA. However, while LGE is useful for predicting outcomes in CA, its ability to quantify myocardial infiltration is limited due to variations in signal patterns and intensities. Consequently, the true capacity of LGE to track changes over time and monitor treatment response remains uncertain. T1 mapping offers potential to address these limitations (34).

Recent research has demonstrated that elevated native myocardial T1 values can effectively predict a worse prognosis in AL CA (38), but not in ATTR CA (53). On the other hand, ECV has been shown to correlate with prognosis in both AL and ATTR CA when adjusted for other independent prognostic factors (38). Additionally, T2 mapping, which measures myocardial edema, is an independent predictor of prognosis in patients with AL CA (54).

Recent studies have demonstrated that ECV results elevated in the earliest phases of the disease highlight the potential role of ECV as an early disease marker (25).

## 4 Diagnostic pitfalls

Despite the high diagnostic value of multimodality imaging, several hallmark findings lack absolute specificity and may lead to diagnostic misclassification if interpreted in isolation. The relative apical sparing pattern on speckle-tracking echocardiography, first described as highly suggestive of cardiac amyloidosis (CA), is typical but not pathognomonic. Similar strain distributions may be observed in hypertensive heart disease, severe aortic stenosis, advanced hypertrophic cardiomyopathy (HCM), and other conditions characterized by basal segment dysfunction (8). Therefore, strain findings should always be integrated with morphological and clinical data.

Important limitations must be acknowledged in the use of bone scintigraphy with 99mTc-labeled tracers; nevertheless, this imaging modality represents a cornerstone for the non-biopsy diagnosis of ATTR cardiac amyloidosis. False negatives may occur in early-stage ATTR, in AL amyloidosis, and in specific hereditary ATTR variants such as Phe84Leu and Ser97Tyr, which are associated with absent or low myocardial tracer uptake (55). Conversely, false-positive uptake has been reported in conditions associated with myocardial injury or increased calcium deposition (56). For this reason, current diagnostic algorithms mandate exclusion of monoclonal gammopathy prior to non-biopsy diagnosis of ATTR-CA (8).

CMR also presents potential pitfalls. Diffuse subendocardial or transmural LGE patterns are highly suggestive of CA but may overlap with advanced HCM or other infiltrative or fibrotic cardiomyopathies (8). Parametric mapping techniques (native T1 and ECV) improve sensitivity for diffuse myocardial infiltration; however, thresholds vary across vendors and field strengths, and universal cut-offs have not been standardized (21).

## 5 Evidence gaps and future directions in multimodality imaging

Despite major advances in multimodality imaging, significant knowledge gaps remain in the diagnostic and prognostic evaluation of HCM and CA.

Most available evidence derives from single-center, retrospective studies with limited external validation and heterogeneous methodologies. Large prospective multicenter studies with standardized imaging protocols are still lacking, limiting the generalizability of diagnostic thresholds and the development of universally accepted algorithms.

Standardization and reproducibility of quantitative imaging biomarkers also remain unresolved. Parameters such as GLS, native T1, ECV, and LGE burden have proven diagnostic and prognostic value, yet they are influenced by vendor-specific software, acquisition techniques, and post-processing variability. Uniform cut-offs for abnormal T1 or ECV values are not universally established, complicating cross-center comparability and integration into risk models.

The incremental prognostic value of advanced imaging markers beyond established clinical risk tools requires further clarification. In HCM, although LGE extent is associated with sudden cardiac death risk, the additive contribution of diffuse fibrosis assessment by T1 mapping remains incompletely defined. In CA, uncertainties persist regarding early detection strategies, particularly in genotype-positive individuals, the variable sensitivity of bone scintigraphy across disease stages and variants (22), and the lack of validated imaging criteria for monitoring response to disease-modifying therapies. Comparative effectiveness and cost-efficiency data are scarce, and the optimal sequencing of echocardiography, CMR, and nuclear imaging has not been tested in outcome-driven frameworks. Emerging approaches including radiomics, quantitative perfusion imaging, hybrid PET/CMR, and artificial intelligence based models for automated phenotyping and risk prediction remain largely investigational and require prospective validation.

Future research should prioritize multicenter prospective studies with harmonized protocols and integration of imaging, genetic, and biomarker data to refine diagnosis, risk stratification, and treatment monitoring in both HCM and CA.

## 6 Conclusion

The differentiation between HCM and CA remains a cornerstone of cardiovascular imaging due to their overlapping phenotypic expressions of left ventricular hypertrophy. Echocardiography and CMR imaging serve as complementary modalities, each providing unique diagnostic insights that enable accurate distinction between these two conditions.

Echocardiography allows assessment of ventricular morphology, diastolic function, and flow dynamics; asymmetric septal hypertrophy, dynamic LV outflow tract obstruction, and SAM are typical of HCM, whereas concentric wall thickening, biatrial enlargement, restrictive filling, and the “apical sparing” pattern on strain imaging are suggestive of CA.

CMR refines the diagnostic process through tissue characterization. In HCM, LGE appears as patchy mid-wall or junctional fibrosis, while in CA it is diffuse and subendocardial or transmural. Mapping techniques further aid differentiation: native T1 and ECV are moderately increased in HCM but markedly elevated in CA due to extracellular amyloid infiltration.

In conclusion, integrating echocardiography and CMR establishes a comprehensive framework for evaluating hypertrophic phenotypes, enhancing diagnostic precision and paving the way for more personalized, imaging-guided management strategies.
