# Supplementary material for: Cardiac imaging in hypertrophic cardiomyopathy and cardiac amyloidosis: a narrative review
Source: Front Cardiovasc Med. 2026 Mar 26;13:1752135. doi: 10.3389/fcvm.2026.1752135 (PMC13062188; doi:10.3389/fcvm.2026.1752135)
Supplement: Supplementary file 1 [file Table1.docx]

Supplementary Table1: ESC Echocardiography and CMR criteria for diagnosis of cardiac amyloidosis.

| **Echocardiography Criteria** | |
| --- | --- |
| Unexplained LV Thickness (>_12 mm) | |
| At least 2 of: -Grade 2 or worse diastolic dysfunction -s^I^, e^I^ and a^I^ waves velocities <5 cm/s at tissue Doppler -Global longitudinal LV strain absolute value < −15% | Score ≥ 8 points: -(IVS wall thickness + PWT)/LVEDD > 0.6 3 points -Doppler E wave/e^I^ wave velocities > 11 1 point -TAPSE ≤ 19 mm 2 points -LV global longitudinal strain absolute value ≤ −13% 1 point -Systolic longitudinal strain apex to base ratio > 2.9 3 points |
| **CMR Criteria** | |
| Characteristic CMR findings (a and b have to be present):  Diffuse subendocardial or transmural LGE  Myocardial nulling preceding or Coinciding with the blood pool (Dark blood pool signal)  ECV ≥ 0.40% (strongly supportive, but not essential/diagnostic) | |
